# Seroprevalence of antibodies against chikungunya virus in Singapore resident adult population

**DOI:** 10.1371/journal.pntd.0006163

**Published:** 2017-12-27

**Authors:** Li Wei Ang, Yiu Wing Kam, Cui Lin, Prabha Unny Krishnan, Joanne Tay, Lee Ching Ng, Lyn James, Vernon J. M. Lee, Kee Tai Goh, Lisa F. P. Ng, Raymond T. P. Lin

**Affiliations:** 1 Public Health Group, Ministry of Health, Singapore; 2 Singapore Immunology Network, Agency for Science, Technology and Research (A*STAR), Singapore; 3 Department of Laboratory Medicine, Tan Tock Seng Hospital, Singapore; 4 Environmental Health Institute, National Environment Agency, Singapore; 5 Saw Swee Hock School of Public Health, National University of Singapore, Singapore; Duke-NUS GMS, SINGAPORE

## Abstract

**Objectives:**

We determined the seroprevalence of chikungunya virus (CHIKV) infection in the adult resident population in Singapore following local outbreaks of chikungunya fever (CHIKF) in 2008–2009.

**Methods:**

Our cross-sectional study involved residual sera from 3,293 adults aged 18–79 years who had participated in the National Health Survey in 2010. Sera were tested for IgG antibodies against CHIKV and dengue virus (DENV) and neutralizing antibodies against CHIKV.

**Results:**

The prevalence of CHIKV-neutralizing antibodies among Singapore residents aged 18–79 years was 1.9% (95% confidence interval: 1.4%– 2.3%). The CHIKV seroprevalence was highest in the elderly aged 70–79 years at 11.5%, followed by those aged 30–39 years at 3.1%. Men had significantly higher CHIKV seroprevalence than women (2.5% versus 1.3%, *p* = 0.01). Among the three main ethnic groups, Indians had the highest seroprevalence (3.5%) compared to Chinese (1.6%) and Malays (0.7%) (*p* = 0.02 and *p* = 0.01, respectively). Multivariable logistic regression identified adults aged 30–39 years and 70–79 years, men, those of Indian ethnicity and ethnic minority groups, and residence on ground floor of public and private housing apartments as factors that were significantly associated with a higher likelihood of exposure to CHIKV. The overall prevalence of anti-DENV IgG antibodies was 56.8% (95% CI: 55.1%– 58.5%), while 1.5% (95% CI: 1.1%– 2.0%) of adults possessed both neutralizing antibodies against CHIKV and IgG antibodies against DENV.

**Conclusions:**

Singapore remains highly susceptible to CHIKV infection. There is a need to maintain a high degree of vigilance through disease surveillance and vector control. Findings from such serological study, when conducted on a regular periodic basis, could supplement surveillance to provide insights on CHIKV circulation in at-risk population.

## Author summary

The prevalence of neutralizing antibodies against chikungunya virus (CHIKV) was low at 1.9% among resident adults in Singapore after local outbreaks in 2008–2009. Adults aged 30–39 years and 70–79 years, men, those of Indian ethnicity and ethnic minority groups, and residence on ground floor of public and private housing apartments were significantly associated with a higher likelihood of exposure to CHIKV.

## Introduction

Chikungunya fever (CHIKF) has re-emerged as an important mosquito-borne disease caused by the Chikungunya virus (CHIKV), an *Alphavirus* belonging to the *Togaviridae* family [1], and transmitted by two main vectors, *Aedes aegypti* and *Aedes albopictus *in the urban cycle [2]. It is characterized by fever, joint pain, headache and myalgia [3]. The disease was first described during an outbreak in southern Tanzania in 1952 [3,4]. Since then, CHIKV outbreaks had been identified in countries in Africa, Asia, Europe, and the Indian and Pacific Oceans [5]. In late 2013, the first evidence of local CHIKV transmission in the Americas emerged when France reported two laboratory-confirmed autochthonous cases in the French part of the Caribbean island of St Martin [6]. Subsequently, local transmission has been identified in 45 countries or territories throughout the Americas, and more than 2.9 million suspected and confirmed cases and 296 deaths have been reported to the Pan American Health Organization from affected areas as of late July 2016 [5,7].

In Asia, CHIKV was first isolated in Bangkok, Thailand, in 1958, and outbreaks of CHIKF have been reported since the 1960s [8,9]. More recent reports of CHIKF outbreaks in Southeast Asia include those of Indonesia in 2001–2003 [10], Malaysia in 2006–2009 [11,12], Thailand in 2008–2009 [13] and Singapore in 2008–2009 [14]. While the earlier outbreaks were associated with the Asian genotype, the recent resurgence was associated with the East/Central/South African (ECSA) genotype [15], which had also caused the preceding outbreaks in the Indian Ocean islands in 2005 [16], India in 2006–2008 [17] and Sri Lanka in 2006–2007 [18–20].

**Fig 1:**
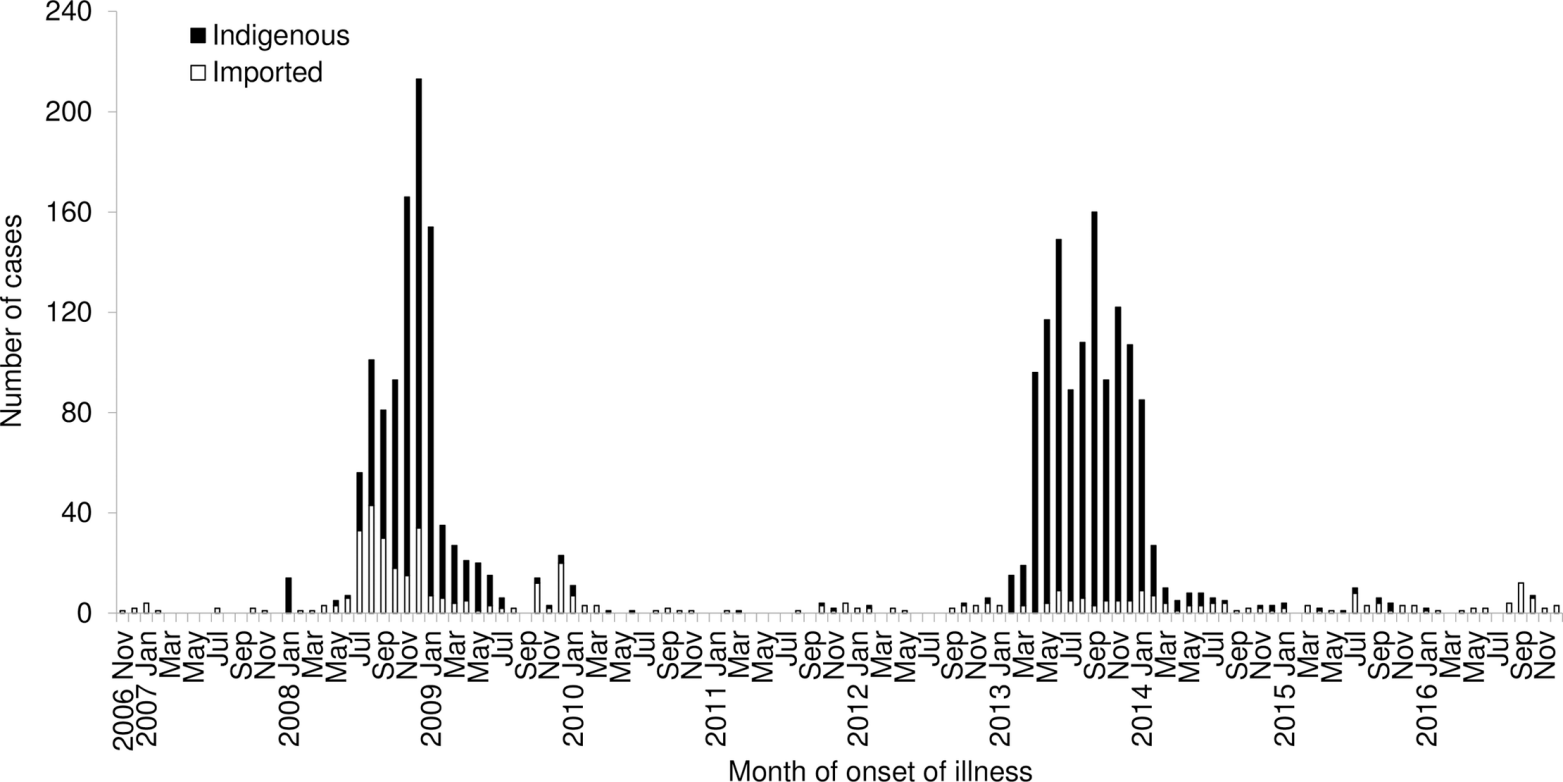
Number of cases of chikungunya fever (CHIKF) notified by month of onset of illness, Singapore, 2006 to 2016.

In Singapore, a tropical city-state, dengue is endemic with all four dengue virus (DENV) serotypes in circulation [21,22]. In response to the regional resurgence of CHIKF and to prevent its introduction into the country, an active laboratory-based surveillance system to detect CHIKV infection was established in late 2006 [23]. General practitioners were requested to consider Chikungunya as a diagnosis when dengue was suspected, and blood samples found to be negative for DENV by polymerase chain reaction (PCR) were routinely tested for CHIKV by PCR and serology. Sporadic imported cases were detected in November 2006. The first confirmed indigenous case infected with ECSA genotype was reported in a 13-year-old Taiwanese student who returned home from Singapore on November 20, 2006 [24]. A localized outbreak of 13 cases, which occurred in an *Aedes aegypti* predominant urban area, was rapidly contained from January to February 2008 (Fig 1) [25]. However, larger outbreaks subsequently occurred from July 2008 to January 2009 in other rural and suburban areas where *Aedes albopictus* was the predominant vector (Fig 1) [14]. The local transmission was attributed to the introduction of a mutated ECSA CHIKV with A226V substitution in the E1 gene [19,22]. E2-I211T substitution was also observed in CHIKV isolates from CHIKF-suspected sera used for full genome sequencing [19,20]. These two strains have been associated with efficient transmission by *Aedes albopictus* [26]. With aggressive vector control measures, the outbreak was finally brought under control in 2009. A total of 1,072 laboratory-confirmed CHIKF cases (260 imported and 812 indigenous) were reported between 2006 and 2009. In 2010, only 26 sporadic laboratory-confirmed cases were reported with 76.9% classified as imported cases [27].

There has been limited information on the seroepidemiology of CHIKV in Singapore and in many countries in South-east Asia. In a serosurvey conducted in Singapore among 531 healthy young adults aged 18–29 years in 2002–2003, two (0.4%) tested positive for IgG antibodies against CHIKV [28]. To assess the impact of the introduction into and spread of CHIKV in Singapore, we undertook a comprehensive serological study to determine its prevalence in the adult resident population.

## Methods

### Study population

We used residual sera from the National Health Survey (NHS) in 2010. The NHS 2010 was a population-based cross-sectional survey conducted by the Ministry of Health to determine the prevalence of major non-communicable diseases and their associated risk factors among Singapore adult residents (Singapore citizens and permanent residents) [29]. Selection of the general population was by a combination of disproportionate stratified sampling and systematic sampling. The survey fieldwork was carried out from 17 March to 13 June 2010 in six sites geographically distributed across the country. A total of 4,337 Singapore residents aged 18–79 years participated in the survey, giving a response rate of 57.7%. Only sera from NHS participants who had given informed consent to allow use of their residual sera for further research were included. Ethical approval was given by the Institutional Review Board Ethics Committee of the Health Promotion Board, Singapore (006/2010).

Residual sera from 3,293 (75.9%) of NHS respondents with sufficient amount leftover were tested. All samples analyzed were anonymized. The socio-demographic profile of these survey respondents in our study and the Singapore resident population aged 18–79 years was found to be similar [30].

### Laboratory assays

All residual serum samples were first tested for IgG antibodies against CHIKV and DENV by anti-enzyme-linked immunosorbent assay (ELISA) using commercial test kits (EUROIMMUN, Germany) according to manufacturer's recommended procedure. Titres ≥ 20 RU/mL were considered to be reactive for both tests.

Samples tested positive for CHIKV IgG antibodies were further evaluated for CHIKV-specific neutralizing antibodies using plaque reduction neutralization tests (PRNT). Neutralizing activity of antibodies from human sera samples were tested in duplicates and analyzed by immunofluorescence-based cell infection assay in HEK 293T cells. CHIKV was mixed at a MOI of 10 with diluted (1:1000), heat-inactivated human sera and incubated for two hours at 37°C with gentle agitation (350 rpm). Virus-antibody mixtures were then added to HEK 293T cells seeded in 96-well plates and incubated for 1.5 hours at 37°C. Medium was removed, and cells were replenished with Dulbecco’s modified Eagle’s medium supplied with 10% FBS and incubated for six hours at 37°C. Live cells were determined by staining with a Live/Dead determination dye (Invitrogen) for 20 minutes according to the manufacturer’s protocol, before fixation with 4% paraformaldehyde and immunofluorescence staining. Cells were permeabilized with PBS containing 0.1% Tween-20, 0.1% Triton X-100, 3% BSA, 5% FBS and incubated for 30 minutes at room temperature. Cells were stained with mouse antibody recognizing CHIKV antigen [31] diluted in PBS for one hour at room temperature. This was followed by incubation with goat anti-mouse secondary antibody conjugated to Alexa Fluor 488 (Invitrogen) for one hour at room temperature. Data were acquired using MACSQuant Analyzer (Miltenyi Biotec) and results were analyzed by the FlowJo v10 software (FlowJo, LLC).

Percentage of infection was calculated according to the equation [% infection = 100 x (% infection from neutralization group/% infection from virus infection group)]. In this study, healthy donors lacking anti-CHIKV antibodies were included as negative controls, and infection ≤85% indicated presence of neutralizing activity to CHIKV [32,33]. Strong CHIKV-specific neutralizing activity was defined as ≤50% of 293T cells were infected by CHIKV post-incubation with the sera, moderate as >50% to 75% and weak as >75% to 85%.

Testing of residual sera for IgG antibodies and neutralizing antibodies against CHIKV was approved by National Healthcare Group's Domain Specific Review Board, Singapore (B/2015/01124).

### Data analysis

To ensure that the characteristics of the NHS 2010 sample conformed to that of the general population, post-stratification weights were computed based on the age, gender, ethnic group and dwelling type attributes of the Singapore resident population. The overall sample weight was the product of weights for unequal probability of selection and non-response from the household enumeration exercise and survey fieldwork, respectively, and post-stratification weight.

The chi-square test or Fisher’s exact test, where appropriate, was used to test for group differences. Crude odds ratios (cOR) and adjusted odds ratios (aOR) with their 95% confidence intervals (CI) were estimated using univariable and multivariable logistic regression models. Listwise deletion was used for missing data of independent variables in the models. Multivariable logistic regression was used to determine independent factors associated with seropositivity, using forward stepwise selection based on maximum partial likelihood estimation with *p* < 0.20 for entry of variables and *p* < 0.05 for removal of variables. All *p* values reported were two-sided and statistical significance was taken as *p* < 0.05. Statistical analyses were performed using SPSS software, version 23 (Armonk, NY: IBM Corp., USA).

## Results

CHIKV IgG was detected in 71 (2.2%, 95% CI: 1.7%– 2.7%) out of 3,293 survey respondents. Of these 71, 61 had CHIKV-specific neutralizing antibodies–the overall prevalence was 1.9% (95% CI: 1.4%– 2.3%).

Compared with seronegative adults, a higher proportion of those tested seropositive were of age 70–79 years, men, ethnic minority groups categorized under ‘others’, retirees, and resided on ground floor of public housing apartments, private flats and condominiums (Table 1).

**Table 1:** Distribution (%) of socio-demographic characteristics according to status of CHIKV-neutralizing antibodies of respondents from National Health Survey, Singapore, 2010.

Socio-demographic characteristics		Serostatus of CHIKV-neutralizing antibodies					Total	
		Seropositive			Seronegative			
		No. (%)			No.	(%)	No.	(%)
*All*		61	(100)		3,232	(100)	3,293	(100)
*Age group*								
	18–29	3	(4.9)		661	(20.5)	664	(20.2)
	30–39	21	(34.4)		661	(20.5)	682	(20.7)
	40–49	6	(9.8)		724	(22.4)	730	(22.2)
	50–59	7	(11.5)		677	(20.9)	684	(20.8)
	60–69	2	(3.3)		339	(10.5)	341	(10.3)
	70–79	22	(36.1)		170	(5.3)	192	(5.8)
*Gender*								
	Male	36	(59.0)		1,384	(42.8)	1,420	(43.1)
	Female	25	(41.0)		1,848	(57.2)	1,873	(56.9)
*Residency*								
	Singapore citizens	47	(77.0)		2,783	(86.3)	2,830	(85.9)
	Permanent residents ^a^	14	(23.0)		443	(13.7)	457	(13.9)
*Ethnic Group*								
	Chinese	39	(63.9)		2,393	(74.0)	2,432	(73.8)
	Malay	3	(4.9)		417	(12.9)	420	(12.8)
	Indian	11	(18.0)		305	(9.4)	316	(9.6)
	Others ^b^	8	(13.1)		117	(3.6)	125	(3.8)
*Main work status over last 12 months* ^*^								
	Working	31	(50.8)		2177	(67.4)	2,208	(67.1)
	Student / National service	1	(1.6)		221	(6.8)	222	(6.7)
	Homemaker/housewife	13	(21.3)		516	(16.0)	529	(16.1)
	Retired	16	(26.2)		215	(6.7)	231	(7.0)
	Unemployed / Unknown	0	(0.0)		96	(3.0)	96	(2.9)
*Type of residential premises*								
	Landed residential property ^c^	4	(6.6)		158	(4.9)	162	(4.9)
	Public housing apartment	54	(88.5)		2729	(84.4)	2,783	(84.5)
	Private flat and condominium	3	(4.9)		326	(10.1)	329	(10.0)
	Others ^d^	0	(0.0)		19	(0.6)	19	(0.6)
*Floor level of residential premises*								
	Landed residential property and others	4	(6.6)		177	(5.5)	181	(5.5)
	Public housing apartment, private flat and condominium							
	Ground	6	(9.8)		97	(3.0)	103	(3.1)
	2^nd^ to 9^th^ floor	38	(62.3)		2022	(62.6)	2,060	(62.6)
	10^th^ floor or higher	13	(21.3)		936	(29.0)	949	(28.8)

* Numbers do not add up to 3,293 due to non-response, such as refusals. There were missing data for 7 individuals tested negative for CHIKV-neutralizing antibodies.

^a^ Singapore permanent residents refer to non-citizens who have been granted permanent residence in Singapore.

^b^ Ethnic group of “Others” comprises all persons other than Chinese, Malays and Indians. They include Eurasians, Caucasians, Japanese, Filipino, Vietnamese, etc.

^c^ Refers to bungalow/ detached house, semi-detached house and terrace house.

^d^ Refers to temporary residences and dormitories.

The seroprevalence was highest in the elderly aged 70–79 years at 11.5%, followed by those aged 30–39 years at 3.1% (Table 2). Men had significantly higher seroprevalence than women (2.5% versus 1.3%, *p* = 0.01). Among the three main ethnic groups, those of Indian ethnicity had a higher seroprevalence (3.5%) compared to that of Chinese (1.6%) (*p* = 0.02) and Malays (0.7%) (*p* = 0.01). The seroprevalence was highest in the ethnic minority groups categorized as ‘others’ (6.4%), which comprises Eurasians, Caucasians, Japanese, Filipino, Vietnamese, etc. The seroprevalence was also highest among retirees at 6.9%. Adults staying on landed residential properties had the highest seroprevalence at 2.5%. Among the adults living in housing apartments, residents on the ground floor had higher seroprevalence than those residing on second or higher levels (*p* = 0.002).

**Table 2:** Seroprevalence (%) and odds ratios for presence of CHIKV-neutralizing antibodies according to socio-demographic characteristics of respondents from National Health Survey, Singapore, 2010.

Socio-demographics		% sero-positive	Univariable analysis			Multivariable analysis*		
			cOR	(95% CI)	*p*-value	aOR	(95% CI)	*p*-value
*Age group (years)*					<0.0005			<0.0005
	18–29	0.5	1.00	Referent		1.00	Referent	
	30–39	3.1	7.31	(2.13–25.08)	0.002	6.58	(1.90–22.80)	0.003
	40–49	0.8	1.93	(0.48–7.83)	0.359	1.89	(0.46–7.73)	0.374
	50–59	1.0	2.34	(0.59–9.25)	0.226	2.51	(0.63–10.02)	0.193
	60–69	0.6	1.53	(0.27–8.72)	0.633	1.68	(0.29–9.68)	0.561
	70–79	11.5	28.23	(8.18–97.47)	<0.0005	34.28	(9.74–120.73)	<0.0005
*Gender*					0.011			0.008
	Male	2.5	1.96	(1.17–3.29)	0.011	2.07	(1.21–3.56)	0.008
	Female	1.3	1.00	Referent		1.00	Referent	
*Residency*					0.039			
	Singapore citizen	1.7	1.00	Referent				
	Permanent resident	3.1	1.89	(1.03–3.47)	0.039			
*Ethnic Group*					<0.0005			0.001
	Chinese	1.6	1.00	Referent		1.00	Referent	
	Malay	0.7	0.39	(0.11–1.35)	0.136	0.36	(0.10–1.28)	0.114
	Indian	3.5	2.10	(1.05–4.18)	0.035	2.14	(1.03–4.43)	0.041
	Others	6.4	4.11	(1.87–9.00)	<0.0005	4.02	(1.70–9.51)	0.002
*Main work status over last 12 months*					<0.0005			
	Working	1.4	1.00	Referent				
	Student / National serviceman	0.5	0.18	(0.01–2.48)	0.200			
	Homemaker	2.5	1.71	(0.88–3.30)	0.113			
	Retired	6.9	5.19	(2.80–9.64)	<0.0005			
	Unemployed / Unknown	0.0	0.13	(0.001–13.88)	0.391			
*Type of residential premises*					0.558			
	Landed residential property and others	2.5	1.00	Referent				
	Public housing apartment	1.9	0.86	(0.30–2.53)	0.788			
	Private flat and condominium	0.9	0.36	(0.07–1.76)	0.207			
	Others	0.0	0.00	-	0.998			
*Floor level of residential premises*					0.011			0.006
	Landed residential property	2.2	1.52	(0.47–4.92)	0.488	1.49	(0.44–5.09)	0.521
	Public and private apartment							
	Ground	5.8	4.99	(1.89–13.15)	0.001	6.55	(2.30–18.72)	<0.0005
	2^nd^ to 9^th^ floor	1.8	1.38	(0.73–2.63)	0.321	1.72	(0.88–3.34)	0.113
	10^th^ floor or higher	1.4	1.00	Referent		1.00	Referent	

cOR, crude odds ratio; aOR: adjusted odds ratio; CI, confidence interval.

* Adjusted for age group, gender, ethnic group and floor level of residential premises.

In the multivariable regression model, independent factors significantly associated with seropositivity were age group, gender, ethnic group and floor level of residential premises (Table 2). Compared to the age group of 18–29 years, adults in the age group of 30–39 years [adjusted OR (aOR): 6.58, 95% CI 1.90–22.80] and those aged 70–79 years (aOR: 34.28, 95% CI 9.74–120.73) were more likely to be CHIKV seropositive (Table 2). Men were more likely to be seropositive compared to women with an aOR of 2.07 (95% CI 1.21–3.56). Compared to the Chinese, adults of Indian ethnicity (aOR: 2.14, 95% CI 1.03–4.43) and ethnic minority groups categorized as ‘others’ (aOR: 4.02, 95% CI 1.70–9.51) were significantly associated with a higher likelihood of exposure to CHIKV. Compared to adults residing on the 10^th^ floor or higher levels of housing apartments, those who stayed on the ground floor were at higher odds of being CHIKV seropositive (aOR: 6.55, 95% CI 2.30–18.72).

**Fig 2:**
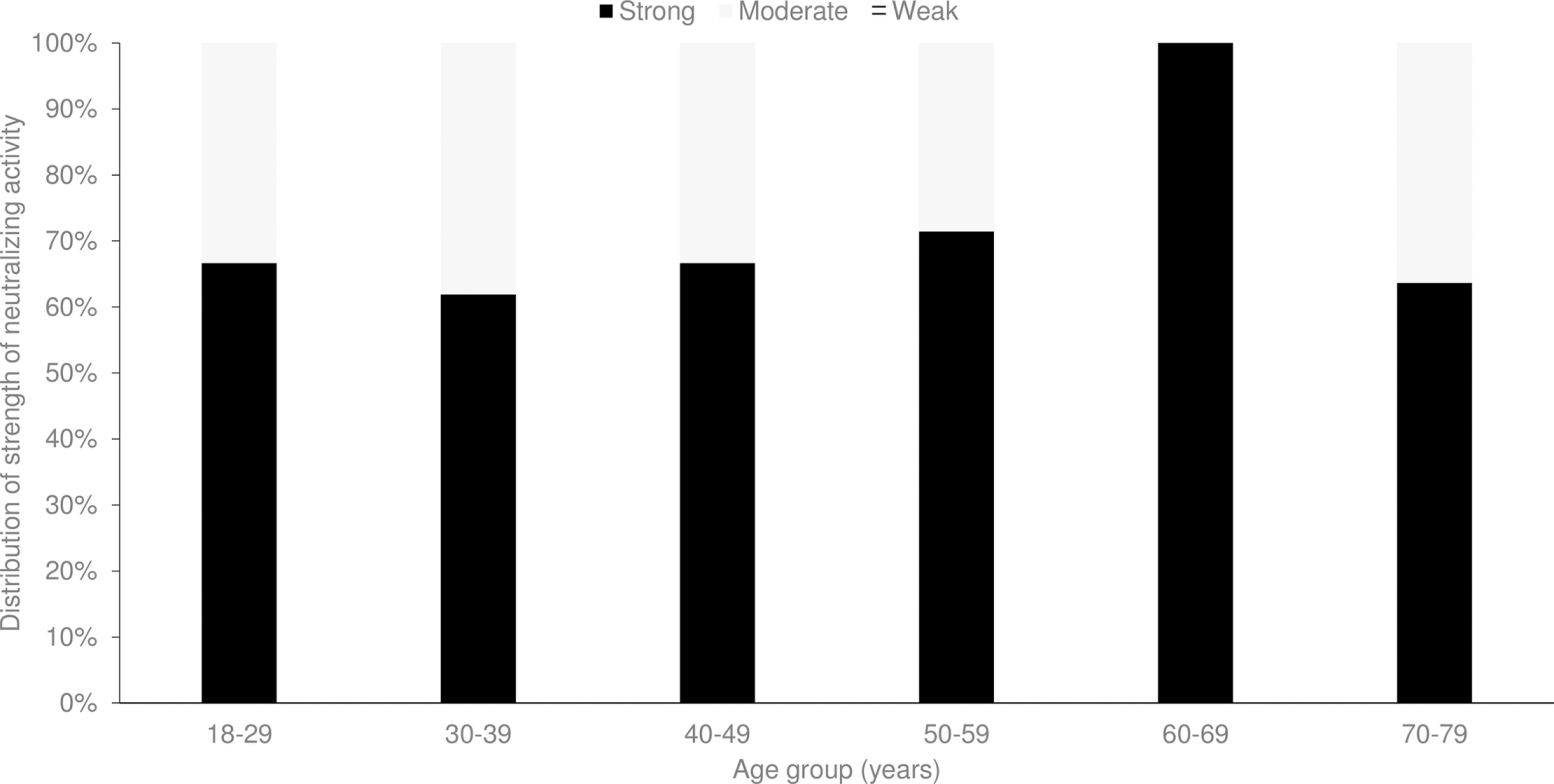
Distribution of strength of chikungunya virus (CHIKV)-specific neutralizing activity by age group among 61 seropositive respondents from National Health Survey, Singapore, 2010.

Among the 61 adults with neutralizing activity to CHIKV, 65.6% had strong neutralizing activity while the rest had moderate neutralizing activity and none had weak neutralizing activity. The proportion of CHIKV seropositive respondents having strong neutralizing activity ranged from 61.9% in the age group of 30–39 years to 100% in the age group of 60–69 years (Fig 2).

The overall prevalence of anti-DENV IgG antibodies was 56.8% (95% CI: 55.1%– 58.5%) [30]. A total of 51 adults (1.5%, 95% CI: 1.1%– 2.0%) had both neutralizing antibodies against CHIKV and IgG antibodies against DENV. Ten adults (0.3%) had neutralizing antibodies against CHIKV only, while 1,821 (55.3%) had IgG antibodies against DENV only.

## Discussion

This was the first nationally representative study to describe the seroepidemiology of CHIKV and determine the magnitude of exposure in the Singapore resident population. Our study showed that about 1.9% of the adults in Singapore had likely been exposed to CHIKV, which was much lower than that of DENV with a prevalence of 56.8% in the same population studied [30]. This is not surprising as dengue has long been endemic in Singapore since the first outbreak reported in 1960 [34,35].

The CHIKV seroprevalence in adults 18–29 years of age (0.5%) after the 2008–2009 outbreaks was similar to that of a smaller study [28] in the same age group (0.4%) five years before these outbreaks (*p* = 0.84). However, the findings of these two studies may not be directly comparable due to differences in laboratory methods. In the earlier serological study in 2002–2003, IgG antibodies were detected using CHIKV-infected cells on teflon-coated glass slide as antigen (Ooi EE, Duke-National University of Singapore Medical School, Singapore, personal communication). Nevertheless, this showed that transmission of infection among young adults in the community was relatively low before and during the outbreak.

The post-outbreak seroprevalence in Singapore was lower compared to that of studies in some countries. In north-eastern Italy, the prevalence of IgG antibody against CHIKV by the indirect immunofluorescence method was 10.2% in 325 residents across all ages surveyed after an outbreak in 2007 [36]. In four Malaysian outbreak-free states, 5.9% in 945 healthy adults aged 35–74 years recruited in 2008 tested positive for CHIKV IgG by ELISA [37]. In Cebu City, the Philippines, 22.0% in 150 individuals across all ages were seropositive for CHIKV from a cross-sectional study using neutralization assays in 1973, while a prospective fever cohort study in 2012 found that 28.3% in 853 residents ≥6 months of age had PRNT titers at baseline indicating a history of CHIKV infection [38]. In central and southern Thailand, a study involving serum samples of 835 individuals aged between 6 months and 60 years obtained in 2014 and analyzed by commercial ELISA test kits found that 26.8% were seropositive for CHIKV, while 24.4% possessed both anti-CHIKV and anti-DENV IgG antibodies [39].

In our study, age group, gender and ethnic group were independently associated with CHIKV seropositivity in the multivariable logistic regression analysis. This generally corresponded with demographic characteristics of reported indigenous laboratory-confirmed cases of CHIKF among Singapore residents, with higher incidence rates in adults aged 30–39 years, men, and ethnic minority groups categorized as ‘others’. The incidence rate of indigenous cases was consistently highest in ethnic minority groups at 7.8 and 4.2 per 100,000 Singapore resident population, followed by Chinese at 6.1 and 3.1 per 100,000 population in 2008 and 2009, respectively [14]. In 2013, another outbreak year, ethnic minority groups also had the highest incidence rate of indigenous cases among Singapore residents (26.9 per 100,000) followed by Chinese (9.4 per 100,000) [40].

CHIKF was known to have swept through Southeast Asia in the 1960s and 1970s [8]. Even though CHIKV was not widely tested then, one patient was incidentally tested positive for CHIKV infection by complement fixation tests and neutralization tests in 1960 during an outbreak of dengue hemorrhagic fever in Singapore [41]. Considering that the vector for dengue and chikungunya is the same *Aedes *mosquitoes, and the *Aedes* house index (percentage of houses infested with *Aedes* larvae/pupae) was in the range of 30–50% at that time, it is likely that Singapore was not spared from disease transmission during the regional CHIKF outbreak, which would have contributed to the highest seroprevalence observed among adults aged 70–79 years. The lower proportion of CHIKV seropositive respondents in this age group having strong neutralizing activity (Fig 2) could be due in part to the decline in neutralizing antibody titers over time.

The gender difference for CHIK seroprevalence in our study had also been observed for dengue-specific IgG prevalence [30]. Higher CHIKV seroprevalence in men was also reported in serological studies in Malaysia [37] and the Indian Ocean island of Mayotte [42]. It has been postulated that the gender differential in the risk of CHIKV infection could be attributed to specific behaviour that results in greater exposure to bites by *Aedes* mosquitoes, and less tendency toward individual protection [36,42,43].

The ethnic difference in seroprevalence could be partly due to exposure during travel to highly endemic countries in the region. The high incidence among Indians corresponded with the highest rate of imported cases among Singapore residents travelling to India during the 2008–2009 outbreaks [14]. Phylogenetic data revealed that the first three reported episodes of local transmission in 2008 were due to three genetically distinct viruses of different geographic origins, suggesting that these episodes may be due to independent importations of CHIKV, most likely from India, Malaysia, and Sri Lanka [22].

The seroprevalence was highest among adults staying on landed residential properties. The incidence rate of indigenous cases of CHIKF and dengue has consistently been the highest for those living on landed residential properties, where there are more potential *Aedes* breeding habitats.

The proportion of adults aged 18–79 years with both neutralizing antibodies against CHIKV and IgG antibodies against DENV detected was low at 1.5%. Among the 1,872 samples tested positive for IgG antibodies against DENV in our serological study, 51 (2.7%) also had neutralizing antibodies against CHIKV. In Thailand, a seroprevalence study to evaluate evidence of past infection against CHIKV and DENV found that 79.2% (661/835) of individuals aged between 6 months and 60 years were DENV-seropositive, of whom 30.9% (204/661) also had IgG antibodies against CHIKV [39].

The first concurrent isolation of CHIKV and dengue type 2 virus (DENV-2) was from a single blood specimen taken from a patient in the acute phase of a dengue-like illness in southern India in 1964 [44]. Since then, a number of cases of co-infection with DENV and CHIKV have been detected in countries/territories such as Angola, Gabon, India, Madagascar, Malaysia, Myanmar, Nigeria, Saint Martin, Singapore, Sri Lanka, Tanzania, Thailand and Yemen; these constitute only 13 out of the 98 countries/territories where both chikungunya and dengue epidemic/endemic transmission have been reported based on literature search conducted until May 2015 for all relevant articles [45]. This included report of one case of imported co-infection of CHIKV and DENV-2 who had returned to Taiwan from Singapore in 2010 [46]. In Singapore, the active laboratory-based surveillance initiated in late 2006 was confined to DENV-negative blood samples for detection of CHIKV, hence cases co-infected with CHIKV and DENV could be potentially missed out.

The vectors for CHIKF and dengue, *Aedes albopictus* and *Aedes aegypti*, are distributed throughout Singapore. *Aedes aegypti* thrives in urban areas while *Aedes albopictus* inhabits in higher proportion in less urbanized areas with greenery in Singapore. A well-established nationwide *Aedes* surveillance and control programme incorporating source reduction, public education, community participation, and law enforcement, has been in place over the last four decades [47]. The overall *Aedes* house index has been maintained at around 1–2%. Despite the aggressive vector control efforts, CHIKF re-emerged in 2013 with a total of 1,059 laboratory-confirmed cases (95.5% indigenous and 4.5% imported), 1.5 times the 718 laboratory-confirmed cases reported in 2008 (Fig 1) [40]. The recurrence of the outbreak of CHIKF coincided with the largest dengue epidemic in the same year, indicating that similar factors may have facilitated the upsurge in the number of cases of these two mosquito-borne diseases in Singapore [48]. Phylogenetic analysis revealed that while locally transmitted CHIKV strains in 2013 formed a monophyletic group within the ECSA genotype, they possessed a signature of two synonymous substitutions (C639T + C816A) in E1 gene, making them a genetically distinct group [40]. These findings, together with the long-term absence of CHIKV transmission on an outbreak scale in Singapore, supported a viral introduction event prior to the establishment of indigenous transmission during the CHIKV outbreak in 2013. Outbreak strains possessed E1-A226V substitution, which further supported the potential role of *Aedes albopictus* as a predominant vector in CHIKV transmission in the 2013 outbreak. Imported virus strains belonged to the ECSA and Asian genotypes and did not possess E1-A226V substitution [40]. The ECSA strains all shared an Indian sub-continent ancestry. While imported strains with the ECSA genotype clustered separately from outbreak strains and were sporadically detected during 2009–2013, those with the Asian genotype were mainly from the Philippines and Indonesia [40].

There are a few limitations in our study. Some of the positive tests for CHIKV infection could have cross-reacted with other arboviruses such as O'nyong-nyong virus (ONNV), Ross River virus (RRV) and Barmah Forest virus (BFV) [33,49–51]. However, we have no data on the prevalence of other alphaviruses in our local population. There has been no or limited data comparing the relative sensitivity or specificity of the available CHIKV diagnostic assays [52]. The low seroprevalence in our study was consistent with sporadic detection of clinical cases. To establish past exposure to CHIKV, we used PRNTs which are deemed to be specific for alphaviruses and serve as the gold standard for confirmation of serological test results [53]. As our study was carried out based on residual sera and not specifically for CHIKV infection, clinical signs and travel history were not recorded.

The re-emergence and spread of CHIKF have been attributed to several factors, including vast immunologically naïve human populations, viral adaption, enhanced efficiency of mosquito transmission, drastic increase in international travel, as well as climate and environmental changes [9,48,54]. As Singapore remains highly susceptible to CHIKV infection, there is a need to maintain a high degree of vigilance through disease surveillance and vector control. Findings from such serological study, when conducted on a regular periodic basis, could supplement surveillance to provide insights on CHIKV circulation and profile of at-risk population.

## Supporting information

S1 Checklist STROBE checklist. (DOCX)
