# Supplementary material for: Seroprevalence of antibodies against chikungunya virus in Singapore resident adult population
Source: PLoS Negl Trop Dis. 2017 Dec 27;11(12):e0006163. doi: 10.1371/journal.pntd.0006163 (PMC5760101; doi:10.1371/journal.pntd.0006163)
Supplement: S1 Checklist — (DOCX) [file pntd.0006163.s001.docx]

STROBE Statement—checklist of items that should be included in reports of observational studies

|  | Item No. | Recommendation | Page  No. | Relevant text from manuscript |
| --- | --- | --- | --- | --- |
| **Title and abstract** | 1 | (*a*) Indicate the study’s design with a commonly used term in the title or the abstract | 2 | Our cross-sectional study involved residual sera from 3,293 adults aged 18-79 years who had participated in the National Health Survey in 2010. |
|  |  | (*b*) Provide in the abstract an informative and balanced summary of what was done and what was found | 2 | Sera were tested for IgG antibodies against CHIKV and DENV, and neutralizing antibodies against CHIKV. The prevalence of CHIKV-neutralizing antibodies among Singapore residents aged 18-79 years was 1.9% (95% confidence interval: 1.4% – 2.3%). 1.5% (95% CI: 1.1% – 2.0%) of adults possessed both neutralizing antibodies against CHIKV and IgG antibodies against DENV. |
| Introduction | | | |  |
| Background/rationale | 2 | Explain the scientific background and rationale for the investigation being reported | 5 | There has been limited information on the sero-epidemiology of CHIKV in Singapore and in many countries in South-east Asia. In a serosurvey conducted in Singapore among 531 healthy young adults aged 18-29 years in 2002-2003, 2 (0.4%) tested positive for IgG antibodies against CHIKV [26]. |
| Objectives | 3 | State specific objectives, including any prespecified hypotheses | 5 | To assess the impact of the introduction into and spread of CHIKV in Singapore, we undertook a comprehensive serological study to determine its prevalence in the adult resident population. |
| Methods | | | |  |
| Study design | 4 | Present key elements of study design early in the paper | 5 | We used residual sera collected from the National Health Survey (NHS) in 2010. |
| Setting | 5 | Describe the setting, locations, and relevant dates, including periods of recruitment, exposure, follow-up, and data collection | 5-6 | The survey fieldwork was carried out from 17 March to 13 June 2010 in six sites geographically distributed across the country. A total of 4,337 Singapore residents aged 18-79 years participated in the survey, giving a response rate of 57.7%. Only sera from NHS participants who had given informed consent to allow use of their residual sera for further research were included. Residual sera from 3,293 (75.9%) of NHS respondents with sufficient amount leftover were tested for CHIKV. All samples analyzed were anonymized. |
| Participants | 6 | (*a*) *Cohort study*—Give the eligibility criteria, and the sources and methods of selection of participants. Describe methods of follow-up  *Case-control study*—Give the eligibility criteria, and the sources and methods of case ascertainment and control selection. Give the rationale for the choice of cases and controls  *Cross-sectional study*—Give the eligibility criteria, and the sources and methods of selection of participants | 5 | The NHS 2010 was a population-based cross-sectional survey conducted by the Ministry of Health to determine the prevalence of major non-communicable diseases and their associated risk factors among Singapore adult residents (Singapore citizens and permanent residents). Selection of the general population was by a combination of disproportionate stratified sampling and systematic sampling. |
|  |  | (*b*) *Cohort study*—For matched studies, give matching criteria and number of exposed and unexposed  *Case-control study*—For matched studies, give matching criteria and the number of controls per case |  |  |
| Variables | 7 | Clearly define all outcomes, exposures, predictors, potential confounders, and effect modifiers. Give diagnostic criteria, if applicable | 6 | Samples tested positive for IgG antibodies were further evaluated for CHIKV-specific neutralizing antibodies using plaque reduction neutralization tests (PRNT). |
| Data sources/ measurement | 8* | For each variable of interest, give sources of data and details of methods of assessment (measurement). Describe comparability of assessment methods if there is more than one group | 6 | Data were acquired using MACSQuant® Analyzer (Miltenyi Biotec) and results were analyzed by the FlowJo v10 software (FlowJo, LLC). |
| Bias | 9 | Describe any efforts to address potential sources of bias | 6 | The socio-demographic profile of these survey respondents in our study and the Singapore resident population aged 18–79 years was found to be similar. |
| Study size | 10 | Explain how the study size was arrived at | 6 | Residual sera from 3,293 (75.9%) of NHS respondents with sufficient amount leftover were tested for CHIKV. |

Continued on next page

| Quantitative variables | 11 | Explain how quantitative variables were handled in the analyses. If applicable, describe which groupings were chosen and why | 7 | Percentage of infection was calculated according to the equation [% infection = 100 x (% infection from neutralization group/% infection from virus infection group)]. In this study, healthy donors lacking anti-CHIKV antibodies were included as negative controls, and infection ≤85% indicated presence of neutralizing activity to CHIKV. Strong CHIKV-specific neutralizing activity was defined as ≤50% of 293T cells were infected by CHIKV post-incubation with the plasma, moderate as >50% to 75% and weak as >75% to 85%. |
| --- | --- | --- | --- | --- |
| Statistical methods | 12 | (*a*) Describe all statistical methods, including those used to control for confounding | 7 | The chi-square test or Fisher’s exact test, where appropriate, was used to test for group differences. Crude odds ratios (cOR) and adjusted odds ratios (aOR) with their 95% confidence intervals (CI) were estimated using univariable and multivariable logistic regression models. |
|  |  | (*b*) Describe any methods used to examine subgroups and interactions | - | - |
|  |  | (*c*) Explain how missing data were addressed | 7 | Listwise deletion was used for missing data of independent variables in the models. |
|  |  | (*d*) *Cohort study*—If applicable, explain how loss to follow-up was addressed  *Case-control study*—If applicable, explain how matching of cases and controls was addressed  *Cross-sectional study*—If applicable, describe analytical methods taking account of sampling strategy | 7 | To ensure that the characteristics of the NHS 2010 sample conformed to that of the general population, post-stratification weights were computed based on the age, gender, ethnic group and dwelling type attributes of the Singapore resident population. The overall sample weight was the product of weights for unequal probability of selection and non-response from the household enumeration exercise and survey fieldwork, respectively, and post-stratification weight. |
|  |  | (*e*) Describe any sensitivity analyses | - | - |
| Results | | | | |
| Participants | 13* | (a) Report numbers of individuals at each stage of study—eg numbers potentially eligible, examined for eligibility, confirmed eligible, included in the study, completing follow-up, and analysed | 8 | CHIKV IgG was detected in 71 (2.2%, 95% CI: 1.7% – 2.7%) out of 3,293 survey respondents. Of these 71, 61 had CHIKV-specific neutralizing antibodies – the overall prevalence was 1.9% (95% CI: 1.4% – 2.3%).  The overall prevalence of anti-DENV IgG antibodies was 56.8% (95% CI: 55.1% – 58.5%) [30]. A total of 51 adults (1.5%, 95% CI: 1.1% – 2.0%) had both neutralizing antibodies against CHIKV and IgG antibodies against DENV. Ten adults (0.3%) had neutralizing antibodies against CHIKV only, while 1,821 (55.3%) had IgG antibodies against DENV only. |
|  |  | (b) Give reasons for non-participation at each stage | - | - |
|  |  | (c) Consider use of a flow diagram | - | - |
| Descriptive data | 14* | (a) Give characteristics of study participants (eg demographic, clinical, social) and information on exposures and potential confounders | 9-10 | Table 1 |
|  |  | (b) Indicate number of participants with missing data for each variable of interest | 10 | Table 1 footnote*:  Numbers do not add up to 3,293 due to non-response, such as refusals. There were missing data for 7 individuals tested negative for CHIKV-neutralizing antibodies. |
|  |  | (c) *Cohort study*—Summarise follow-up time (eg, average and total amount) |  |  |
| Outcome data | 15* | *Cohort study*—Report numbers of outcome events or summary measures over time |  |  |
|  |  | *Case-control study—*Report numbers in each exposure category, or summary measures of exposure |  |  |
|  |  | *Cross-sectional study—*Report numbers of outcome events or summary measures | 8 | CHIKV IgG was detected in 71 (2.2%, 95% CI: 1.7% – 2.7%) out of 3,293 survey respondents.  The overall prevalence of anti-DENV IgG antibodies was 56.8% (95% CI: 55.1% – 58.5%) [30]. A total of 51 adults (1.5%, 95% CI: 1.1% – 2.0%) had both neutralizing antibodies against CHIKV and IgG antibodies against DENV. |
| Main results | 16 | (*a*) Give unadjusted estimates and, if applicable, confounder-adjusted estimates and their precision (eg, 95% confidence interval). Make clear which confounders were adjusted for and why they were included | 8, 12 | CHIKV IgG was detected in 71 (2.2%, 95% CI: 1.7% – 2.7%) out of 3,293 survey respondents.  In the multivariable regression model, independent factors associated with seropositivity were age group, gender, ethnic group and floor level of residential premises (Table 2). |
|  |  | (*b*) Report category boundaries when continuous variables were categorized | 11 | Age group |
|  |  | (*c*) If relevant, consider translating estimates of relative risk into absolute risk for a meaningful time period | - | - |

Continued on next page

| Other analyses | 17 | Report other analyses done—eg analyses of subgroups and interactions, and sensitivity analyses | - | - |
| --- | --- | --- | --- | --- |
| Discussion | | | | |
| Key results | 18 | Summarise key results with reference to study objectives | 13 | Our study showed that about 1.9% of the adults in Singapore had likely been exposed to CHIKV. |
| Limitations | 19 | Discuss limitations of the study, taking into account sources of potential bias or imprecision. Discuss both direction and magnitude of any potential bias | 15 | Some of the positive tests for CHIKV infection could have cross-reacted with other arboviruses. There has been no or limited data comparing the relative sensitivity or specificity of the available CHIKV diagnostic assays. The low seroprevalence in our study was consistent with sporadic detection of clinical cases. To establish past exposure to CHIKV, we used PRNTs which are deemed to be specific for alphaviruses and serve as the gold standard for confirmation of serological test results. As our study was carried out based on residual sera and not specifically for CHIKV infection, clinical signs and travel history were not recorded. |
| Interpretation | 20 | Give a cautious overall interpretation of results considering objectives, limitations, multiplicity of analyses, results from similar studies, and other relevant evidence | 13 | The CHIKV seroprevalence in adults 18-29 years of age (0.5%) after the 2008-2009 outbreaks was similar to that of a smaller study [28] in the same age group (0.4%) five years before these outbreaks (p = 0.84). |
| Generalisability | 21 | Discuss the generalisability (external validity) of the study results | 13,15 | This was the first nationally representative study to describe the seroepidemiology of CHIKV in the Singapore resident population, which provide important information on the exposure of the population to CHIKV.  To establish past exposure to CHIKV, we used PRNTs which are deemed to be specific for alphaviruses and serve as the gold standard for confirmation of serological test results. |
| Other information | |  | | |
| Funding | 22 | Give the source of funding and the role of the funders for the present study and, if applicable, for the original study on which the present article is based | - | - |

*Give information separately for cases and controls in case-control studies and, if applicable, for exposed and unexposed groups in cohort and cross-sectional studies.

**Note:** An Explanation and Elaboration article discusses each checklist item and gives methodological background and published examples of transparent reporting. The STROBE checklist is best used in conjunction with this article (freely available on the Web sites of PLoS Medicine at http://www.plosmedicine.org/, Annals of Internal Medicine at http://www.annals.org/, and Epidemiology at http://www.epidem.com/). Information on the STROBE Initiative is available at www.strobe-statement.org.
